# Extremely Rare Flavonoid Glycosides Identified in the Stems of *Ephedra gerardiana* by HPLC-MS and Their Antioxidant Activity

**DOI:** 10.3390/ijms26073097

**Published:** 2025-03-27

**Authors:** Karolina Szymborska, Rafał Frański, Błażej Gierczyk, Monika Beszterda-Buszczak

**Affiliations:** 1 Faculty of Chemistry, Adam Mickiewicz University, Uniwersytetu Poznańskiego 8, 61-614 Poznań, Poland; karszy20@st.amu.edu.pl (K.S.); hanuman@amu.edu.pl (B.G.); 2 Department of Food Biochemistry and Analysis, Poznań University of Life Sciences, Mazowiecka 48, 60-623 Poznań, Poland; monika.beszterda@gmail.com

**Keywords:** flavonoid glycosides, *Ephedra gerardiana*, mass spectrometry, liquid chromatography, fragmentation patterns, DPPH

## Abstract

The plants of the genus Ephedra are mainly known for the contents of alkaloids; however, it has recently been found that they also contain phenolic constituents that show interesting bioactivities, e.g., antioxidative or antimicrobial. From among the Ephedrae herba, the *Ephedra gerardiana* seems to be relatively poorly researched in terms of flavonoid presence. In this study, on the basis of the results of high-pressure liquid chromatography–mass spectrometry (HPLC-MS) with cone voltage-induced fragmentation analysis, which are discussed in detail, the flavonoid glycosides present in *Ephedra gerardiana* have been identified. Besides the flavonoids typical of the genus Ephedra, e.g., afzelin, herbacetin 7-*O*-glucoside, and vicenin-2, compounds that are very rare in nature have been detected as well, namely the *p*-coumaroyl conjugates of 4′-*O*-methylafzelin and malonyl flavone *C*-glycosides. Therefore, *Ephedra gerardiana* can be regarded as a valuable source of these compounds. Furthermore, the antioxidant activity of the methanol extract indicates that these compounds show potential interesting biological activities.

## 1. Introduction

The plants of the genus Ephedra are mainly known for the occurrence of alkaloids, mainly ephedrine and pseudoephedrine, and a few others, e.g., methylephedrine, norephedrine, or norpseudoephedrine [1,2,3,4,5]. Due to the bioactivity of these compounds, for thousands of years, Ephedrae herba has been used in traditional Chinese herbal medicine. Presently, it is known that Ephedrae herba also contains a variety of interesting phenolic constituents that may exhibit various types of bioactivities, e.g., antioxidative, antimicrobial, and antiproliferative [6].

Among the plants of the genus Ephedra, *Ephedra gerardiana* seems to be relatively poorly researched in terms of flavonoid content [7]. The antioxidant activities and other properties of the various extracts of *Ephedra gerardiana* have been widely studied and are usually accompanied by the determination of total phenolic and/or total flavonoid content (TPC, TFC) [6,8,9,10]. Identification of individual phenolic compounds in *Ephedra gerardiana*, to the best of our knowledge, has rarely been the subject of studies. Ibragic et al. have analyzed these compounds in *Ephedra gerardiana* by using thin-layer chromatography (TLC) followed by gas chromatography–mass spectrometry (GC-MS) with injection port derivatization [6], Osmic et al. by using high-pressure liquid chromatography–diode array detector (HPLC-DAD) [11], and Lu et al. have used the HPLC-MS for this purpose [12]. In order to identify the flavonoid glycosides, the most appropriate and powerful method seems to be HPLC-MS. However, Lu et al., among the 213 metabolites, have not mentioned any flavonoid glycosides (the authors mentioned three flavonoids, namely epicatechin, catechin, and dihydrokaempferol). This paper provides, on the basis of the results of HPLC-MS with cone voltage-induced fragmentation analysis, the first identification of the flavonoid glycosides present in *Ephedra gerardiana*. The obtained results may be regarded as a desirable and valuable supplement to the papers concerning the antioxidant activities of the *Ephedra gerardiana* extracts. As described further in detail, together with the expected compounds, a few very unusual flavonoid glycosides have been detected. Therefore, the obtained results indicate that *Ephedra gerardiana* may show potential interesting biological activities that have not been explored yet.

## 2. Results and Discussion

The identified flavonoid glycosides can be divided into two groups, namely *O*-glycosides and *C*-glycosides. In the hydrolyzed sample, due to the partial hydrolysis of flavonoid *O*-glycosides, three aglycones were detected, and they were identified as kaempferol (rt = 10.2 min), kaempferide (4′-*O*-methylkaempferol, rt = 11.7 min), and herbacetin (rt = 8.5 min), as shown in the Supplementary Materials Figure S1. Therefore, it can be taken for granted that flavonoid *O*-glycosides present in the analyzed methanolic extracts are conjugates of these compounds. The *C*-glycosides were apigenin-6,8-di-*C*-glycosides (hexosides/pentosides) and their malonylated conjugates. The compound identification has been performed on the basis of the detected product ions of X, Y, Z, B, and C types (in the widely accepted nomenclature of glycoconjugates fragmentation [13]). The spectra obtained in the negative ion mode have been more useful since more product ions have been detected. Furthermore, in the positive ion mode, especially for low-abundant compounds, a higher background has been observed (e.g., Figure S3, positive ion mode). For more abundant compounds, the background was negligible (e.g., Figure S6, negative ion mode).

It should be stressed that there are no doubts whether the product ions are really coming from respective [M − H]^−^/[M + H]^+^ parent ions (although fragmentation “in-source” may raise such doubts), as the retention times (RTs) of parent and product ions were carefully checked and they always matched perfectly. The analyses were performed in the fast switch mode; thus, the retention times of positive and negative ions of a given compound also perfectly matched.

### 2.1. Afzelin and Its Conjugates

**Scheme 1:**
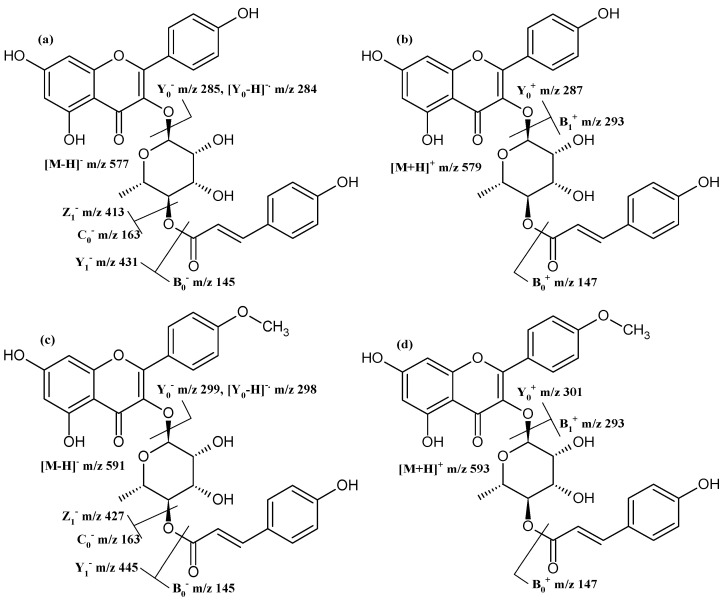
The fragmentation pathways of [M + H]^+^ and [M − H]^−^ ions of (4″-[*E*]-*p*-coumaroyl)afzelin (**a**,**b**) and 4′-O-methyl-(4″-[*E*]-p-coumaroyl)afzelin (**c**,**d**).

The most abundant group of *O*-glycosides includes afzelin (kaempferol 3-*O*-rhamnoside) and its *p*-coumaroyl conjugates, 4′-*O*-methylafzelin (kaempferide 3-*O*-rhamnoside) and its *p*-coumaroyl conjugates. As afzelin and its *p*-coumaroyl conjugates have been identified in various representatives of the genus Ephedra, their occurrence in *Ephedra gerardiana* was expected [14,15,16,17]. The mono-*p*-coumaroyl conjugates contain the *p*-coumaroyl moiety substituted at position 4″ [15,16]. The di-*p*-coumaroyl conjugates contain *p*-coumaroyl substituted at positions 2″ and 4″ [17]. Although in the plants of the genus Ephedra the isomers containing both [*E*]-*p*-coumaroyl and [*Z*]-*p*-coumaroyl moieties have been detected, in this work only one isomer for each *p*-coumaroyl conjugate was detected, and the presence of [*E*]-*p*-coumaroyl moiety was inferred, as the isomers containing [*E*]-*p*-coumaroyl moiety were definitely more abundant than those containing [*Z*]-*p*-coumaroyl moiety in the plants of the genus Ephedra [16,17]. The obtained results of HPLC-MS analysis of afzelin and its conjugates are summarized in Table 1; the respective chromatograms and mass spectra are shown in the supporting information (Figures S2–S7). On the basis of the detected product ions, the fragmentation pathways of [M + H]^+^ and [M − H]^−^ ions have been elucidated (Scheme 1, Schemes S1 and S2 in the Supplementary Material).

It should be stressed that the abundant presence of [Y_0_ − H]^−●^ product ions indicates the glycosylation at position C-3 of the aglycone moiety [18,19,20]. Besides the afzelin and its *p*-coumaroyl conjugates, 4′-*O*-methylafzelin (kaempferide 3-*O*-rhamnoside) and its *p*-coumaroyl conjugates were also detected. Due to the presence of a methyl group on the aglycone moiety, the Y and Z-type ions of 4′-*O*-methylafzelin and its conjugates occur at m/z values by 14 units higher than the analogous ions of afzelin and its conjugates, whereas B and C-type ions correspond to the same m/z values. To the best of our knowledge, the fragmentation pathways of [M − H]^−^/[M + H]^+^ ions of *p*-coumaroyl conjugates of afzelin and *p*-coumaroyl conjugates of 4′-*O*-methylafzelin have not been earlier comprehensively described.

As mentioned above, afzelin and its *p*-coumaroyl conjugates have been earlier identified in various Ephedra species [14,15,16,17], whereas 4′-*O*-methylafzelin has not been earlier identified in the plants of the genus Ephedra (although it has been identified in many other plants [21,22,23]). The *p*-coumaroyl conjugates of 4′-*O*-methylafzelin are extremely rare compounds. To the best of our knowledge, they have been relatively recently identified only in two species, namely in *Lindera akoensis* [24] and *Machilus litseifolia* [25]. *Ephedra gerardiana* can be regarded as the third natural source of *p*-coumaroyl conjugates of 4′-*O*-methylafzelin. It has to be added that these compounds show promising anti-inflammatory activity and have been found to be effective α-glucosidase inhibitors [24,25].

### 2.2. Other Flavonoid O-Glycosides

The obtained results of HPLC-MS analysis of other flavonoid glycosides are summarized in Table 2. The most abundant among the other flavonoid glycosides was herbacetin 7-*O*-glucoside (Figure S8). Herbacetin is an active constituent of various herbs, including Ephedrae herba [26,27]. It is a flavonol, and in nature, the most common glycosylation site of flavonols is position C-3. However, it has been found that the most common glycosylation site of herbacetin is C-7 [14,15,26,28], and rarely C-3 and C-8 [29]. As expected for herbacetin 7-*O*-glucoside, there are abundant Y_0_^+^ and Y_0_^−^ product ions (Figure S8, m/z 303 and 301, respectively). However, even at high cone voltage (CV = 150 V), there is no [Y_0_ − H]^−●^ product ion at m/z 300. It has already been established that the lack of the [Y_0_ − H]^−●^-type ion, or its very low abundance, is a characteristic feature of flavonols and flavones glycosylated at the C-4′ site [18,30,31,32,33]. On the other hand, Ablajan and Tuoheti also have not detected the [Y_0_ − H]^−●^ product ion in the mass spectrum of rhodiosin (herbacetin 7-*O*-glucorhamnoside) and detected a low abundant [Y_0_ − H]^−●^ product ion in the mass spectrum of rhodionin (herbacetin 7-*O*-rhamnoside) [34]. Therefore, although flavonoids 7-*O*-glycosides usually yield [Y_0_ − H]^−●^ product ions, herbacetin 7-*O*-glycosides seem to be the exceptions that do not yield them.

**Figure 1:**
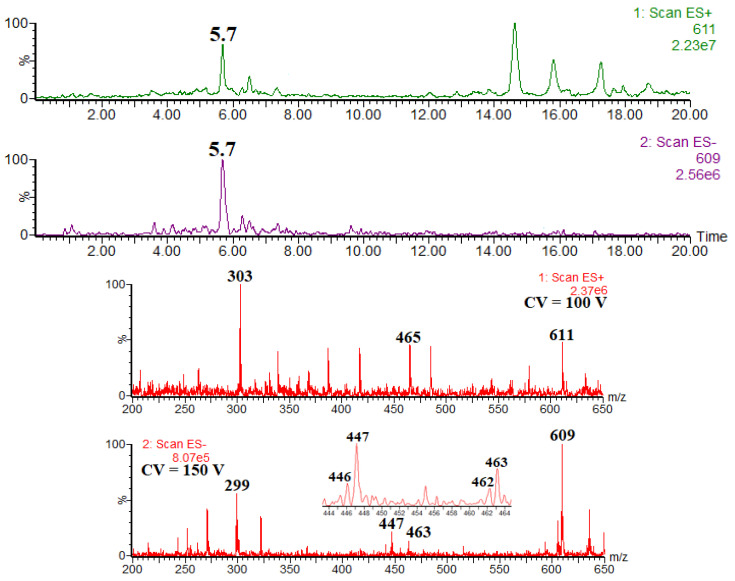
HPLC-MS data obtained for herbacetin-4′-*O*-rhamnoside-7-*O*-glucoside.

Besides the above described, most abundant, kaempferol/herbacetin conjugates, three flavonoid di-glycosides of minor abundances were identified, namely two di-*O*-glycosides, herbacetin-4′-*O*-rhamnoside-7-*O*-glucoside (rt = 5.6 min, Figure 1), kaempferol-4′-*O*-rhamnoside-7-*O*-glucoside (rt = 6.4 min), and one *O*-diglycoside, kaempferol 7-*O*-neohesperidoside (kaempferol 7-*O*-(2″-*O*-rhamnosyl) glucoside, rt = 6.7 min), as summarized in Table 2. The abundant [Y_0_ − 2H]^−^ product ions are characteristic of di-*O*-glycosides and allow their differentiation from *O*-di-glycosides [19,35]. The low abundances of product ions, formed in the negative ion mode due to the homolytic bond cleavage, allow the exclusion of glycosylation at position C-3 [18,19,20]. Since in the positive ion mode, the efficiency of sugar loss from the C-4′ site is higher than that from the C-7 site [36], the loss of rhamnose moiety (loss of mass 146) from [M + H]^+^ ions (Figure 1, Table 2) permitted elucidation of the substitution of rhamnose moiety at C-4′ site in di-*O*-glycosides. The comparable abundances of [Y_0_ − H]^−●^ and [Y_0_]^−^ product ions indicate the C-7 glycosylation site in the kaempferol 7-*O*-neohesperidoside [33] and the low abundant product ion at m/z 449 indicates the rhamnose as a terminal sugar and 1-2 interglycosidic linkage [37].

### 2.3. Apigenin-6,8-di-C-Glycosides and Their Malonylated Conjugates

The fragmentation pathways of flavone *C*-glycosides of [M − H]^−^ ions are more useful and less complicated than that of [M + H]^+^ ions; therefore, only the ESI mass spectra obtained in the negative ion mode are discussed for these compounds (Table 3).

The flavone *C*-glycosides are quite common in plants from the genus Ephedra [14,28,29,38]; therefore, the detection of flavone di-*C*-glycosides in *Ephedra gerardiana* is not surprising. The first identified flavone di-*C*-glycoside was vicenin-2, which is apigenin 6,8-di-*C*-glucoside (MW = 594, Figure S9), probably the most abundant flavonoid di-*C*-glycoside in nature (its isomers, e.g., genistein 6,8-di-C-glucoside or apigenin 6-C-galactoside-8-C-glucoside are also known but are definitely less common [39,40]). The typical fragmentation of *C*-glycosides in the negative ion mode, namely the loss of mass 120 and/or 90 (^0,2^X^−^ and ^0,3^X^−^-type ions, respectively [41]) was observed, as shown in the obtained mass spectrum (Figure S9). Although the relative abundances of product ions formed in CID conditions can vary depending on the instrumental conditions (e.g., collision energy), the ratios of abundances of product ions m/z503/m/z473 as well as m/z383/m/z353 for vicenin-2 are constant and about 0.3 and 0.6, respectively, as reported in many papers [41,42,43,44,45]. As we have noted similar ratios (Figure S9), the identification of vicenin-2 seems to be unambiguous.

Flavonoid di-*C*-glycoside of MW = 564 was detected at rt = 5.4 min (Table 3), and the chromatographic peak shape suggests that we deal with a mixture of isomers (Figure S10). It is understandable that these isomers may have very similar chromatographic properties, resulting in co-elution upon HPLC-MS analysis. It can be taken for granted that this compound (and its isomer) is composed of apigenin, hexose moiety (most probably glucose moiety), and pentose moiety (arabinose or xylose moiety); thus, it can be assigned as apigenin 6,8-di-*C*-hexoside/pentoside. The common flavonoid di-*C*-glycosides of MW = 564 are schaftoside, (apigenin 6-*C*-glucoside-8-*C*-arabinoside), isoschaftoside (apigenin 6-*C*-arabinoside-8-*C*-glucoside), vicenin-1 (apigenin 6-*C*-xyloside-8-*C*-glucoside), and vicenin-3 (apigenin 6-*C*-glucoside-8-*C*-xyloside); however, other isomers have been also mentioned in the literature [44]. Upon collision-induced dissociation of their [M − H]^−^ ions (m/z 563), besides the loss of mass 120 and 90, the loss of mass 60 can also occur (fragmentation of pentose moiety). The identified most abundant isomer (rt = 5.4 min) was schaftoside, as indicated by the low abundant product ion at m/z 503 (loss of mass 60) and by the obtained ratios of the other product ions, namely m/z383/m/z353 = 0.8 and m/z473/m/z443 = 0.7 (Figure S10). These values are in good agreement with those obtained elsewhere [46,47,48].

**Figure 2:**
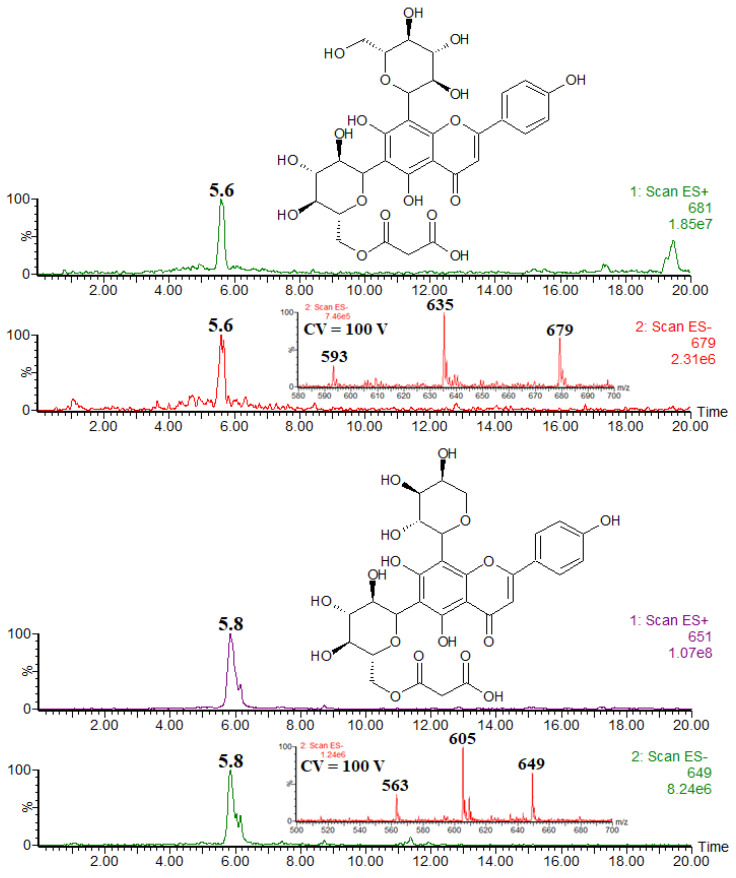
HPLC-MS data and plausible structures of malonyl flavonoid di-*C*-glycosides.

Besides the above-described flavone di-*C*-glycosides, we have detected their malonyl conjugates (Table 3). Insertion of malonyl moiety increases the molecular weight by 86; thus, they have molecular masses of 680 and 650, as shown in Figure 2. Analogously, as for flavone di-*C*-glycosides, as indicated by the chromatographic peak shapes, there is only one malonyl conjugate of vicenin-2, and most probably the mixture of isomers of malonyl conjugates of schaftoside (the isomers have very similar chromatographic properties). It was found that the characteristic feature of the identified malonyl flavonoid di-*C*-glycosides, in the negative ion mode, is the loss of CO_2_ (loss of mass 44) followed by the loss of the CH_2_CO molecule (the loss of mass 42), as shown in Figure 2.

Although malonyl flavonoid *O*-glycosides are quite common in nature, e.g., malonyl isoflavone glucosides occur in soy [49], and kaempferol 3-*O*-malonylglucoside and its conjugates occur in leek [50]. Quercetin 3-*O*-malonylglucoside occurs in okra [51]. Malonyl flavonoid *C*-glycosides are extremely rare since, so far, to the best of our knowledge, there have been only three reports of their detection [52,53,54]. The health-benefiting properties of flavonoid *C*-glycosides are already well known [55,56,57], and it may be of interest to find out how the presence of a malonyl moiety affects these properties. *Ephedra gerardiana* may be a valuable source of malonyl flavonoid C-glycosides to perform the respective studies.

It should be stressed that malonyl conjugates have not been detected at all in the acidified extract analyzed in this work, which indicates that they are unstable (elusive) molecules, which may be the reason why malonyl flavonoid *C*-glycosides have hardly ever been reported so far.

### 2.4. Relative Abundances of the Identified Flavonoid Glycosides

As the standards of some of the identified compounds are unavailable, i.e., those of the *p*-coumaroyl conjugates of 4′-*O*-methylafzelin and malonyl flavonoid *C*-glycosides, quantitative determination of the analyzed compounds would be very difficult. However, in order to evaluate at least semi-quantitively the relative abundances of the identified flavonoid glycosides, the chromatographic peak areas of [M + H]^+^ and [M − H]^−^ ions (obtained at a low cone voltage in order to avoid fragmentation) were compared with that of catechin (catechin is a constituent of all vascular plants and has been detected as well, see the Supplementary Material, Figure S11). Of course, we are aware of the weakness of this approach since the ESI responses of the different analyzed compounds are surely not identical. On the other hand, we deal with one type of compound (flavonoid glycosides); therefore, it is reasonable that their ESI responses are comparable. None of the compounds contain substituents, which would significantly affect its base/acid properties (significantly affecting the abundances of [M + H]^+^ and [M − H]^−^ ions).

**Figure 3:**
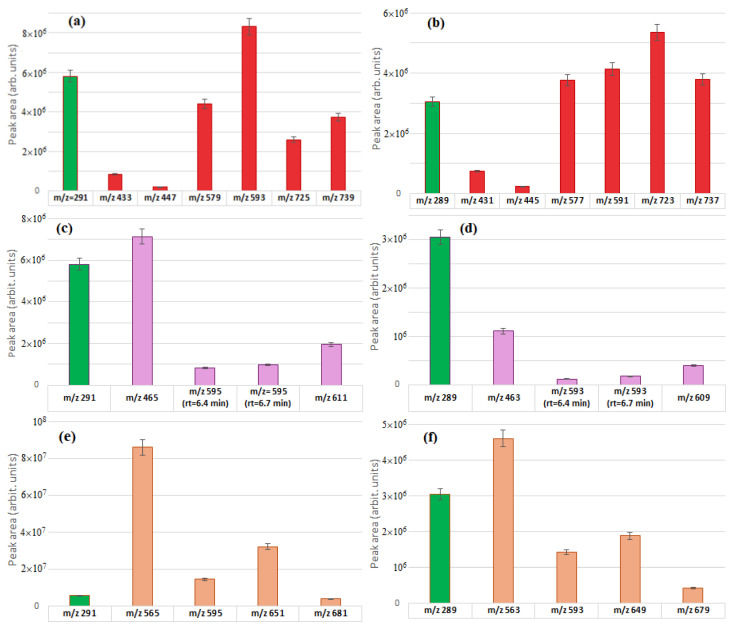
Comparison of the chromatographic peak areas of [M + H]^+^ and [M − H]^−^ ions of identified compounds with that of catechin (m/z 291 and 289). Afzelin and its conjugates, (**a**,**b**); the other flavonoid *O*-glycosides, (**c**,**d**); di-*C*-glycosides and their malonylated conjugates, (**e**,**f**).

As shown in Figure 3, the chromatographic peak areas obtained in both positive and negative ion modes indicate that the contribution of the rarely found compounds identified in this study to the total flavonoid glycoside content is quite significant. According to the data obtained in the positive ion mode, 4′-*O*-methyl-(4″-[*E*]-*p*-coumaroyl)afzelin (m/z 593) is the most abundant compound among the afzelin conjugates (Figure 3a), and according to the data obtained in the negative ion mode, (m/z 591) is the second most abundant compound. The malonyl conjugate(s) of schaftoside is the second most abundant compound among the di-*C*-glycosides, as indicated by the data obtained in the positive (m/z 651) and negative (m/z 649) ion modes (Figure 3e,f).

### 2.5. Antioxidant Activity and Total Flavonoid Content

The antioxidant activities of various Ephedra gerardiana extracts have been widely studied by using a common DPPH assay (scavenging effect of 1,1-diphenyl-2-picrylhydrazyl) [6,8,9,10]. Although the DPPH assay is probably the most commonly used method of phenolic antioxidant activity evaluation, a large number of DPPH assay reports may contain mistakes in the determination of Absolute/Relative Inhibitory Concentrations (A/R IC_50_), as described in detail by de Menezes et al. [58]. Therefore, the antioxidant activity was determined according to the procedure described by de Menezes et al. [58]. The AIC_50_ of the studied extract was determined using the DPPH method, yielding a value of 588 ± 15 g/mol DPPH, while for the reference, i.e., L-ascorbic acid, the value was 51.4 ± 0.2 g/mol DPPH (RIC_50_ = 0.292 ± 0.001). The obtained total flavonoid content was 69 ± 2 mg of rutin equivalent per 1 g of dry mass of the analyzed extract, i.e., ~7%. Therefore, the antioxidant activity of flavonoids present in the *Ephedra gerardiana* seems to be significant.

## 3. Materials and Methods

### 3.1. Preparation of the Extract for HPLC-MS Analysis

Fresh plant material, i.e., stems of *Ephedra gerardiana* Wall. ex Klotzsch and Garcke var. sikkimensis Stapf., was collected in November at the Botanical Garden of Adam Mickiewicz University in Poznań. A portion of 2 g was extracted with 10 mL of pure CH_3_OH. The sample was shaken at 500 rpm for 30 min (Vortex 3, IKA-Werke GmbH, Staufen, Germany), sonicated, and filtered through PTFE syringe filters with a pore size of 0.45 μm (Macherey-Nagel GmbH, Düren, Germany). Prior to the HPLC/ESI-MS analysis, the sample was further diluted at 1:1 in pure CH_3_OH (stored at 5 °C). In order to perform extraction and partial hydrolysis of the extracted compounds simultaneously, a 5% methanolic solution of HCl (30%, ultra-pure, Chem-Lab) was used instead of pure CH_3_OH. The further procedure was the same as above.

### 3.2. HPLC-MS Analysis

The HPLC-MS analyses were performed using a Waters Arc HPLC pump and a Waters SQD mass spectrometer (single quadrupole-type instrument equipped with electrospray ionization (ESI) source, Z-spray, Milford, MA, USA). The software used was MassLynx V4.2 SCN1046 (Milford, MA, USA). Using an autosampler, the sample solutions were injected into the XTerra^®^ MS C18 column (5 μm, 150 mm × 3 mm i.d.). The injection volume was 10 µL. The solutions were analyzed by using a linear gradient of CH_3_CN-H_2_O with a flow rate of 0.5 mL/min. The gradient started from 0% CH_3_CN to 95% H_2_O with 5% of a 10% solution of formic acid in water, reaching 95% CH_3_CN after 15 min, and the latter concentration was maintained for 10 min. The ESI mass spectra were recorded in the m/z range 100–1000, in positive and negative modes simultaneously (during the HPLC/ESI-MS analyses, the mass spectrometer was switched in the fast mode between the positive and negative ion modes). The ESI source potentials were as follows: capillary, 3 kV; lens, 0.5 V; extractor, 4 V; cone voltage, 50, 100, or 150 V. This parameter has the greatest impact on the full scan mass spectra recorded. An increase in this parameter leads to the so-called “in-source” fragmentation/dissociation, but a too-low cone voltage may cause a decrease in sensitivity. The source temperature was 120 °C, and the desolvation temperature was 300 °C. Nitrogen was used as the nebulizing and desolvating gas at flow rates of 100 and 300 L h^−1^, respectively. In order to evaluate the relative abundances of identified compounds, the HPLC-MS analyses have been performed in triplicate, and the relative standard deviation did not exceed 5%.

### 3.3. Preparation of the Extract for Determination of Antioxidant Activity and Total Flavonoid Content

A portion of 1.0 g of dried, powdered plant material was suspended in CH_3_OH (10 mL) and left at room temperature for 12 h. After that, the extract was sonicated for 1 h and filtered through a PTFE membrane (0.45 μm). The dry content in the prepared extract was 11.3 ± 0.2 mg mL^−1^.

### 3.4. Antioxidant Activity Assay

The antioxidant activity was determined according to the method described by de Menezes et al. [58]. A solution of L-ascorbic acid in CH_3_OH (8.5 mM) was used as a standard. DPPH solution in CH_3_OH (0.148 mM) was mixed with an appropriate volume of the studied solution or reference solution, adjusted to 2 mL with CH_3_OH, and left for 30 min in the dark at room temperature. After that, the absorbance at 516 nm was measured. The AIC_50_ and RIC_50_ values were determined based on four independent measurements.

### 3.5. Total Flavonoid Content

Portions of 0.1 mL of AlCl_3_ (10% m/m in H_2_O) and 0.1 mL of CH_3_COOK (1 M in water) were added to 1.8 mL of CH_3_OH. Then, 10 μL of the studied solution or standard solution was added. The obtained mixture was left for 1 min, and the absorbance was measured at 415 nm [59]. A calibration curve was prepared for rutin (2 ng–1 μg; R2 = 0.995). Determination of Total Flavonoid Content in the studied extract was performed in triplicate.

## 4. Conclusions

Summing up, a number of flavonoid glycosides have been identified in the methanolic extract of *Ephedra gerardiana* on the basis of the characteristic fragmentation patterns obtained by HPLC-MS analysis with the cone voltage-induced “in-source” fragmentation. Among the identified compounds, there are the expected ones, e.g., herbacetin 7-*O*-glucoside or vicenin-2, which have well-known biological activities. Along with the expected ones, a few compounds that are extremely rare in nature have been identified as well, namely 4′-*O*-methyl-(4″-[*E*]-*p*-coumaroyl)afzelin, 4′-*O*-methyl-(2″,4″-di-[*E*]-*p*-coumaroyl)afzelin, and malonyl conjugates vicenin-2 and schaftoside. The relative chromatographic peak areas indicate the high relative abundances of the rare compounds among the whole identified flavonoid glycosides. Therefore, *Ephedra gerardiana* can be regarded as a valuable source of the *p*-coumaroyl conjugates of 4′-*O*-methylafzelin and malonyl flavone *C*-glycosides. Furthermore, the high antioxidant activity of the methanol extract indicates that the compounds show potential interesting biological activities that should be investigated.

## Figures and Tables

**Table 1:** The results of HPLC-MS analysis obtained for afzelin and its conjugates.

Compound	rt	[M − H]^−^ [M + H]^+^	Product Ions (Type)
afzelin	7.6	431 433	285 (Y_0_^−^), 284 ([Y_0_ − H]^−•^), 287 (Y_0_^+^)
(4″-[*E*]-*p*-coumaroyl)afzelin	10.2	577 579	431 (Y_1_^−^), 413 (Z_1_^−^), 285 (Y_0_^−^), 284 ([Y_0_ − H]^−•^), 163 (C_0_^−^), 145 (B_0_^−^) 293 (B_1_^+^), 287 (Y_0_^+^), 147 (B_0_^+^)
(2″,4″-di-[*E*]-*p*-coumaroyl)afzelin	12.2	723 725	577 (Y_1_^−^), 559 (Z_1_^−^), 437 (B_1_^−^), 285 (Y_0_^−^), 284 ([Y_0_ − H]^−•^), 163 (C_0_^−^), 145 (B_0_^−^) 439 (B_1_^+^), 147 (B_0_^+^)
4′-*O*-methylafzelin	8.5	445 447	299 (Y_0_^−^), 298 ([Y_0_ − H]^−•^) 301 (Y_0_^+^)
4′-*O*-methyl-(4″-[*E*]-*p*-coumaroyl)afzelin	11.1	591 593	445 (Y_1_^−^), 427 (Z_1_^−^), 299 (Y_0_^−^), 298 ([Y_0_ − H]^−•^), 163 (C_0_^−^), 145 (B_0_^−^) 293 (B_1_^+^), 301 (Y_0_^+^), 147 (B_0_^+^)
4′-*O*-methyl-(2″,4″-di-[*E*]-*p*-coumaroyl)afzelin	13.0	737 739	591 (Y_1_^−^), 573 (Z_1_^−^), 437 (B_1_^−^), 299 (Y_0_^−^), 298 ([Y_0_ − H]^−•^), 163 (C_0_^−^), 145 (B_0_^−^) 439 (B_1_^+^), 147 (B_0_^+^)

**Table 2:** The results of HPLC-MS analysis obtained for flavonoid glycosides.

Compound	rt	[M − H]^−^ [M + H]^+^	Product Ions (Type)
herbacetin 7-*O*-glucoside	6.8	463 465	301 (Y_0_^−^) 303 (Y_0_^+^)
herbacetin-4′-*O*-rhamnoside-7-O-glucoside	5.7	609 611	463 (Y^4′^_0_^−^), 447 (Y^7^_0_^−^), 301 (Y_0_^−^), 299 ([Y_0_ − 2H]^−^) 465 (Y^4′^_0_^+^), 303 (Y_0_^+^)
kaempferol-4′-*O*-rhamnoside-7-*O*-glucoside	6.4	593 595	447 (Y^4′^_0_^−^), 431 (Y^7^_0_^−^), 285 (Y_0_^−^), 283 ([Y_0_ −2H]^−^) 449 (Y^4′^_0_^+^), 287 (Y_0_^+^)
kaempferol 7-*O*-neohesperidoside	6.7	593 595	285 (Y_0_^−^), 284 ([Y_0_ − H]^−•^) 449 (Y_1_^+^, low abundance), 287 (Y_0_+)

**Table 3:** The results of HPLC-MS analysis obtained for apigenin-6,8-di-*C*-glycosides and their malonylated conjugates.

Compound	rt	[M − H]^−^	Product Ions (Type)
apigenin 6,8-di-*C*-glucoside (vicenin-2)	5.1	593	503 (^0,3^X^−^), 473 (^0,2^X^−^), 383 (^0,3/0,2^X^−^), 353 (^0.2/0.2^X^−^)
apigenin 6-*C*-glucoside-8-*C*-arabinoside (schaftoside) and its isomer	5.4	563	503 (^0,3^X^−^), 473 (^0,3^X^−^), 443 (^0,2^X^−^), 383 (^0,3/0,2^X^−^), 353 (^0,3/0,2^X^−^)
malonyl conjugate of vicenin-2	5.6	679	635 ([M – H − CO_2_]^−^), 593 ([M – H − CO_2_ − CH_2_CO]^−^)
malonyl conjugate of schaftoside and its isomer	5.8	649	605 ([M – H − CO_2_]^−^), 563 ([M − H-CO_2_ − CH_2_CO]^−^)

## Data Availability

The data presented in this study are available in this article.

## Supplementary Materials

The following supporting information can be downloaded at https://www.mdpi.com/article/10.3390/ijms26073097/s1.
