# Supplementary material for: Extremely Rare Flavonoid Glycosides Identified in the Stems of Ephedra gerardiana by HPLC-MS and Their Antioxidant Activity
Source: Int J Mol Sci. 2025 Mar 27;26(7):3097. doi: 10.3390/ijms26073097 (PMC11989176; doi:10.3390/ijms26073097)
Supplement: Supplementary file 1 [file ijms-26-03097-s001.zip › ijms-3538100-supplementary.pdf]

Extremely rare flavonoid glycosides identified in the stems of *Ephedra gerardiana* by HPLC-MS and their antioxidant activity

Table of Contents:

Figure S1. HPLC-MS identification of the free aglycones in the hydrolyzed sample (by comparison with the HPLC-MS analysis of the aglycone standards; kaempferol - rt = 10.2 min, kaempferide (4′-O-methylkaempferol) - rt = 11.7 min, herbacetin - rt = 8.5. …………………2

Figure S2. Single ion chromatograms of m/z 433 and 431, and ESI mass spectra of afzelin. ..3

Figure S3. Single ion chromatograms of m/z 447 and 445, and ESI mass spectra of 4'-O-methylafzelin. Because this compound is present in low amount, the high background is observed, especially in the positive ion mode. …………………………………………………...4

Figure S4. Single ion chromatograms of m/z 579 and 577, and ESI mass spectra of (4″-E-*p*-coumaroyl)afzelin. ……………………………………………..5

Figure S5. Single ion chromatograms of m/z 593 and 591, and ESI mass spectra of 4'-O-methyl-(4″-E-*p*-coumaroyl)afzelin. …………………………………………….....................6

Figure S6. Single ion chromatograms of m/z 725 and 723, and ESI mass spectra of (2″,4″-di-E-*p*-coumaroyl)afzelin. ……………………………………………..................................7

Figure S7. Single ion chromatograms of m/z 739 and 737, and ESI mass spectra of 4'-O-methyl-(2″,4″-di-E-*p*-coumaroyl)afzelin. …………………………………………….........8

Scheme S1. The fragmentation pathways of $[M+H]^+$ and $[M-H]^-$ ions of afzelin (**a** and **b**) and 4'-*O*-methylafzelin (**c** and **d**). ……………………………………………..........................9

Scheme S2. The fragmentation pathways of $[M+H]^+$ and $[M-H]^-$ ions of (2″,4″-di-[*E*]-*p*-coumaroyl)afzelin (**a** and **b**) and 4'-*O*-methyl-(2″,4″-di-[*E*]-*p*-coumaroyl)afzelin (**c** and **d**). ..10

Figure S8. HPLC-MS data and observed fragmentation pathways of herbacetin 7-*O*-glucoside. …11

Figure S9. HPLC-MS data of the compound identified as vicenin-2. ………………………12

Figure S10. HPLC-MS data of the compound identified as schaftoside. ……………………12

Figure S11. HPLC-MS identification of the catechin. ………………………………………13

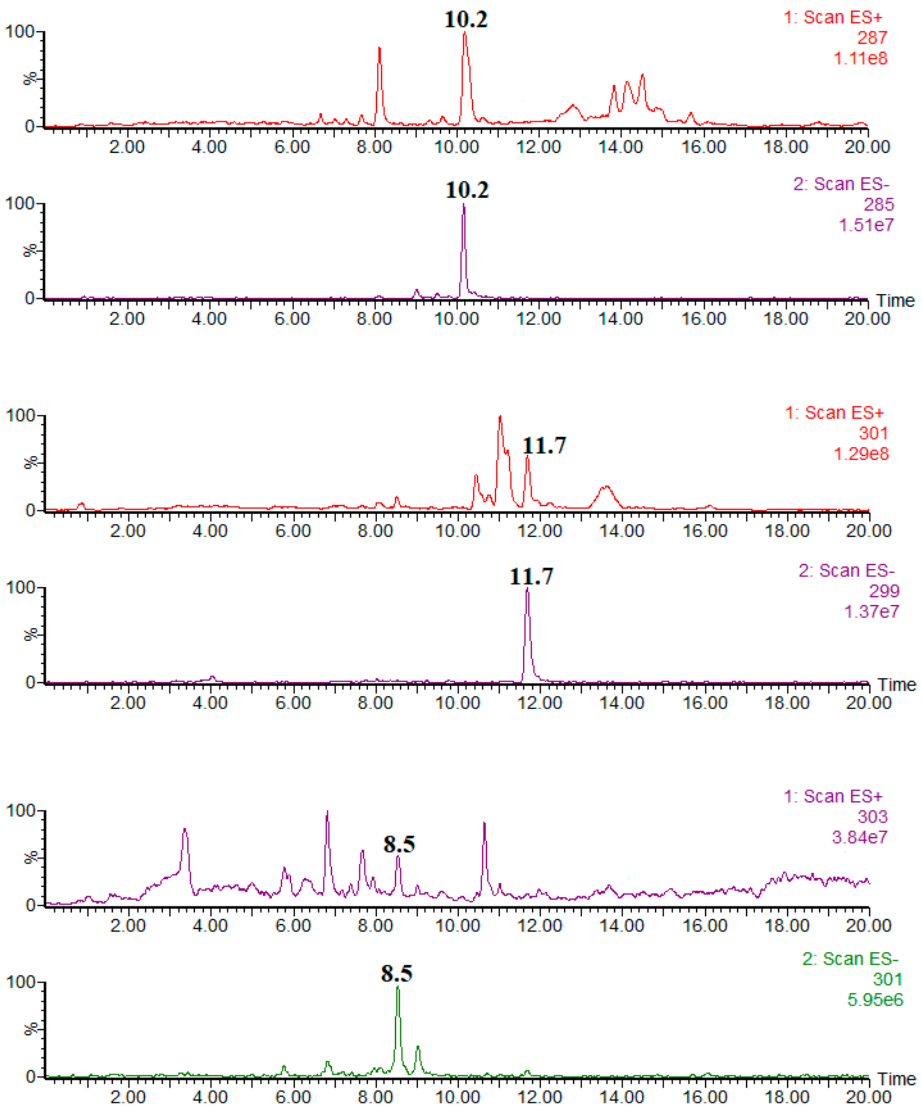

Figure S1. HPLC-MS identification of the free aglycones in the hydrolyzed sample (by comparison with the HPLC-MS analysis of the aglycone standards; kaempferol - rt = 10.2 min, kaempferide (4′-O-methylkaempferol) - rt = 11.7 min, herbacetin - rt = 8.5.

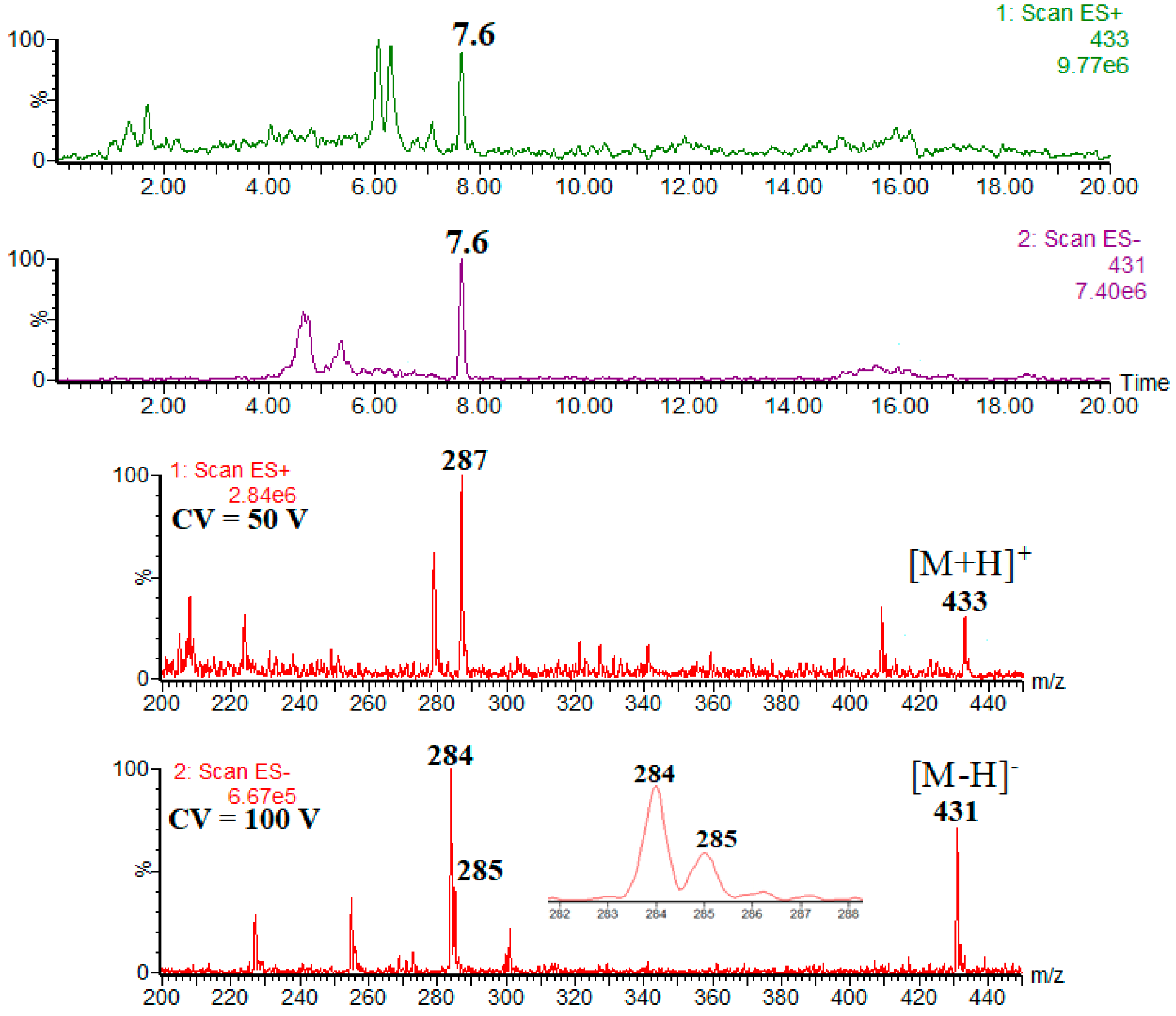

Figure S2. Single ion chromatograms of m/z 433 and 431, and ESI mass spectra of afzelin.

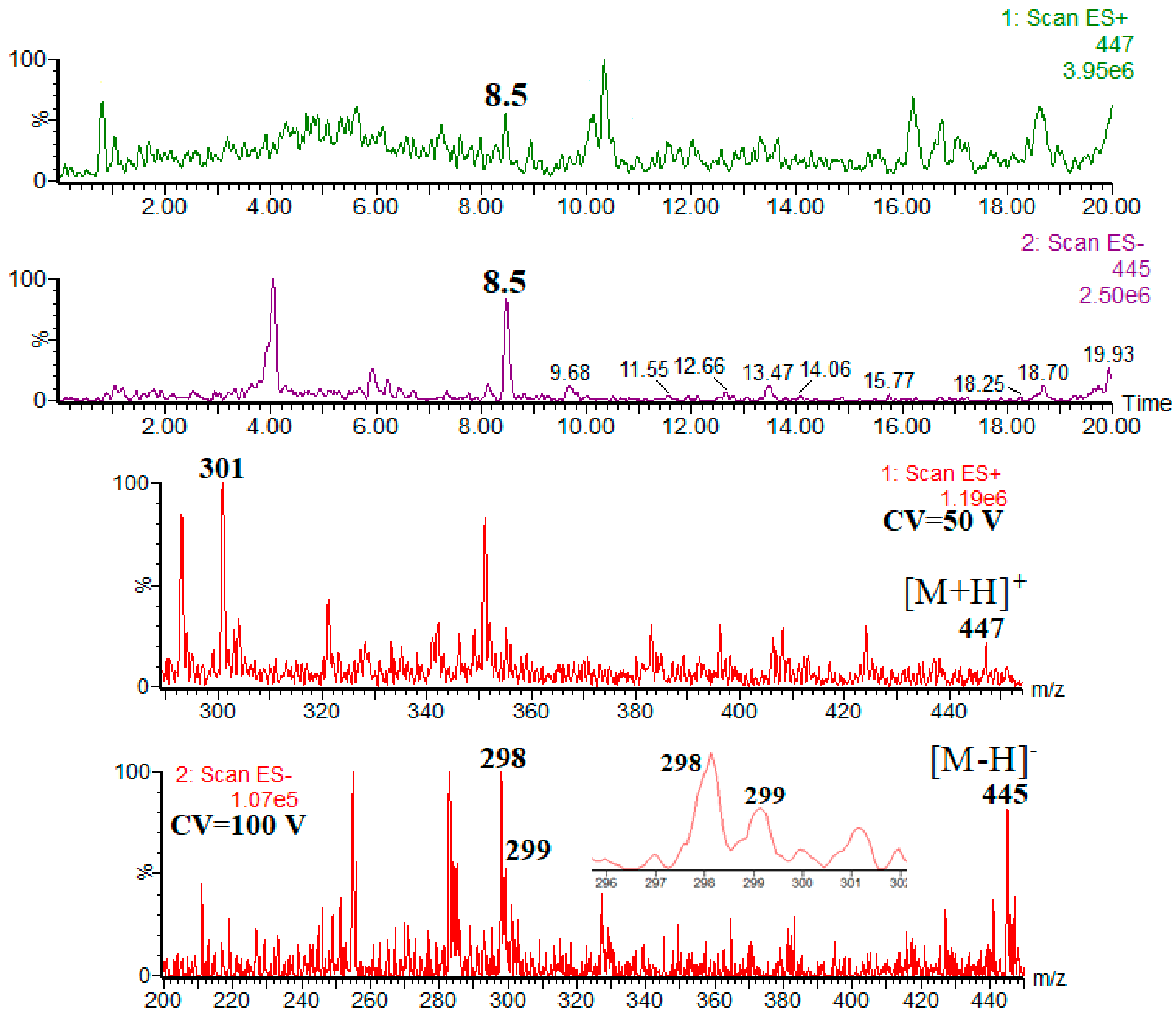

Figure S3. Single ion chromatograms of m/z 447 and 445, and ESI mass spectra of 4'-O-methylafzelin. Because this compound is present in low amount, the high background is observed, especially in the positive ion mode.

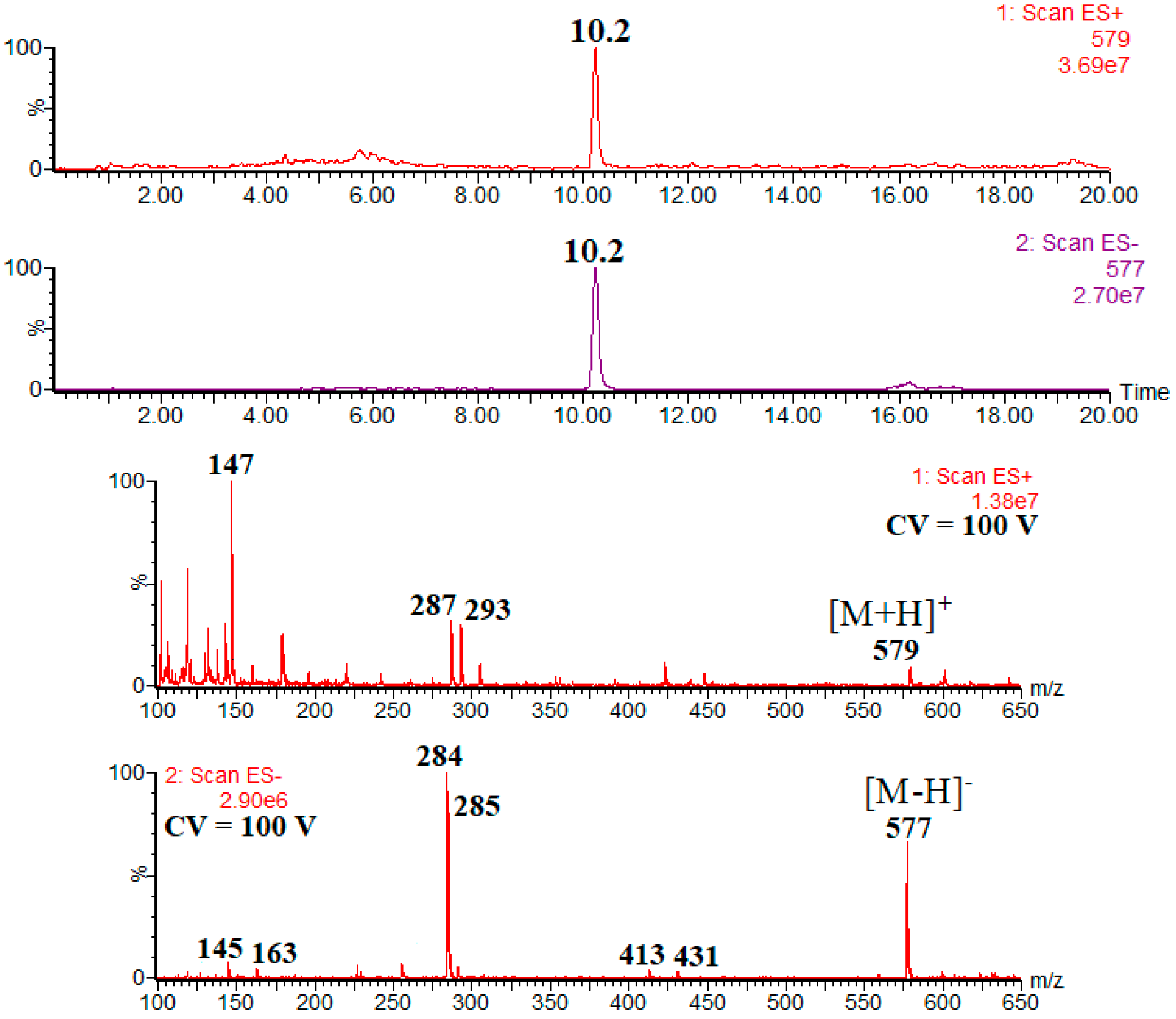

Figure S4. Single ion chromatograms of m/z 579 and 577, and ESI mass spectra of (4″-E-*p*-coumaroyl)afzelin.

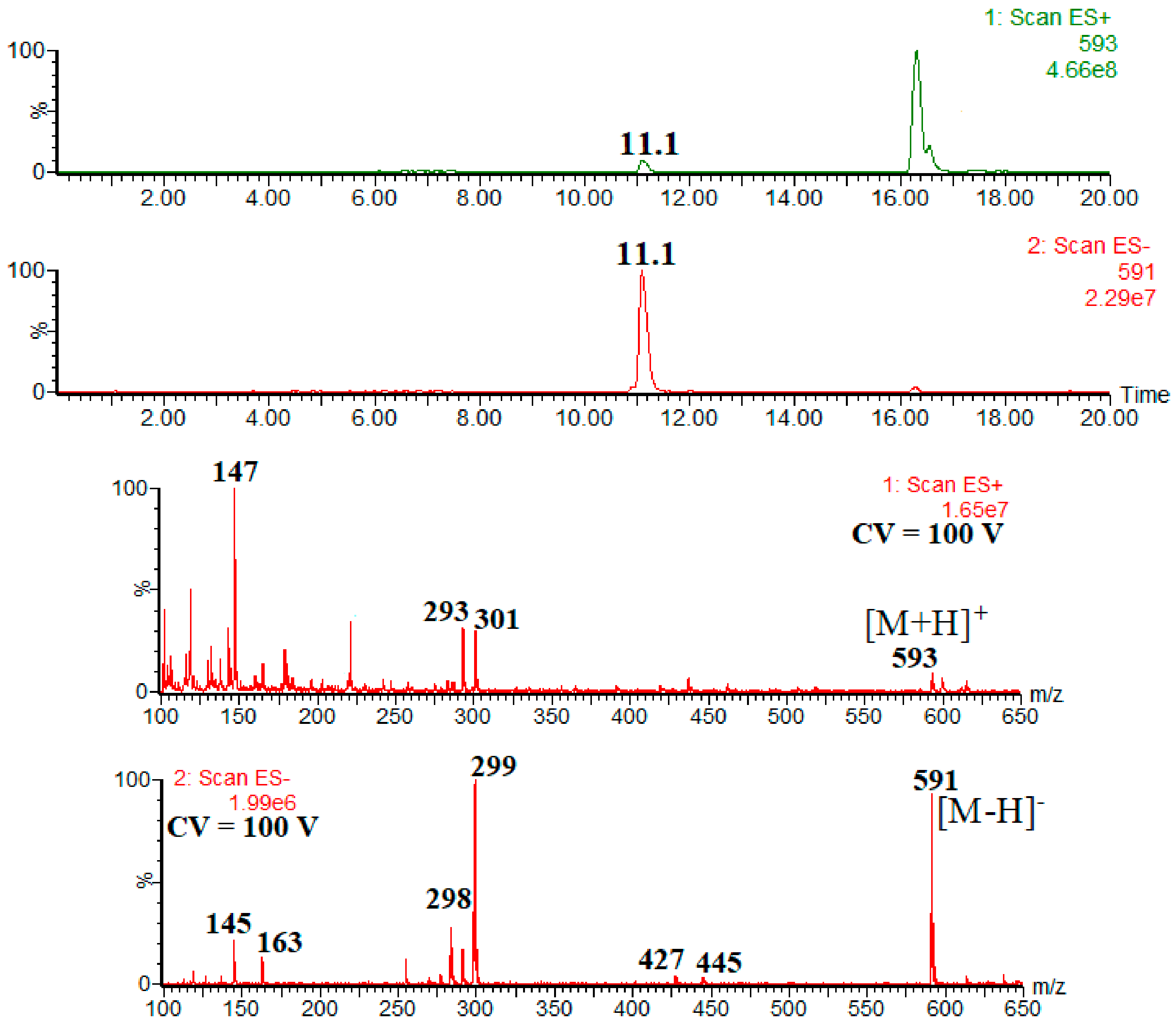

Figure S5. Single ion chromatograms of m/z 593 and 591, and ESI mass spectra of 4’-O-methyl-(4″-E-*p*-coumaroyl)afzelin.

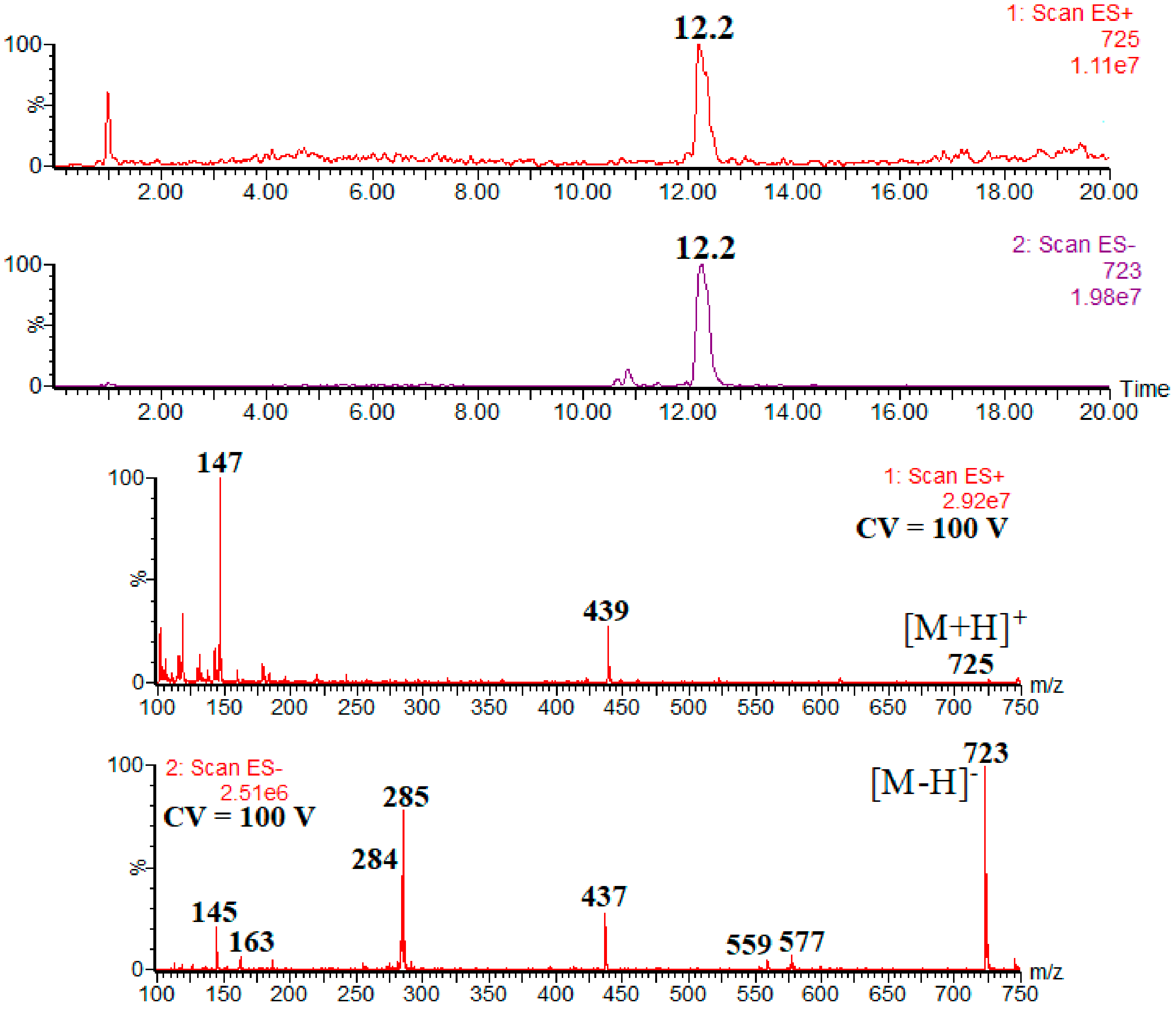

Figure S6. Single ion chromatograms of m/z 725 and 723, and ESI mass spectra of (2″,4″-di-E-*p*-coumaroyl)afzelin.

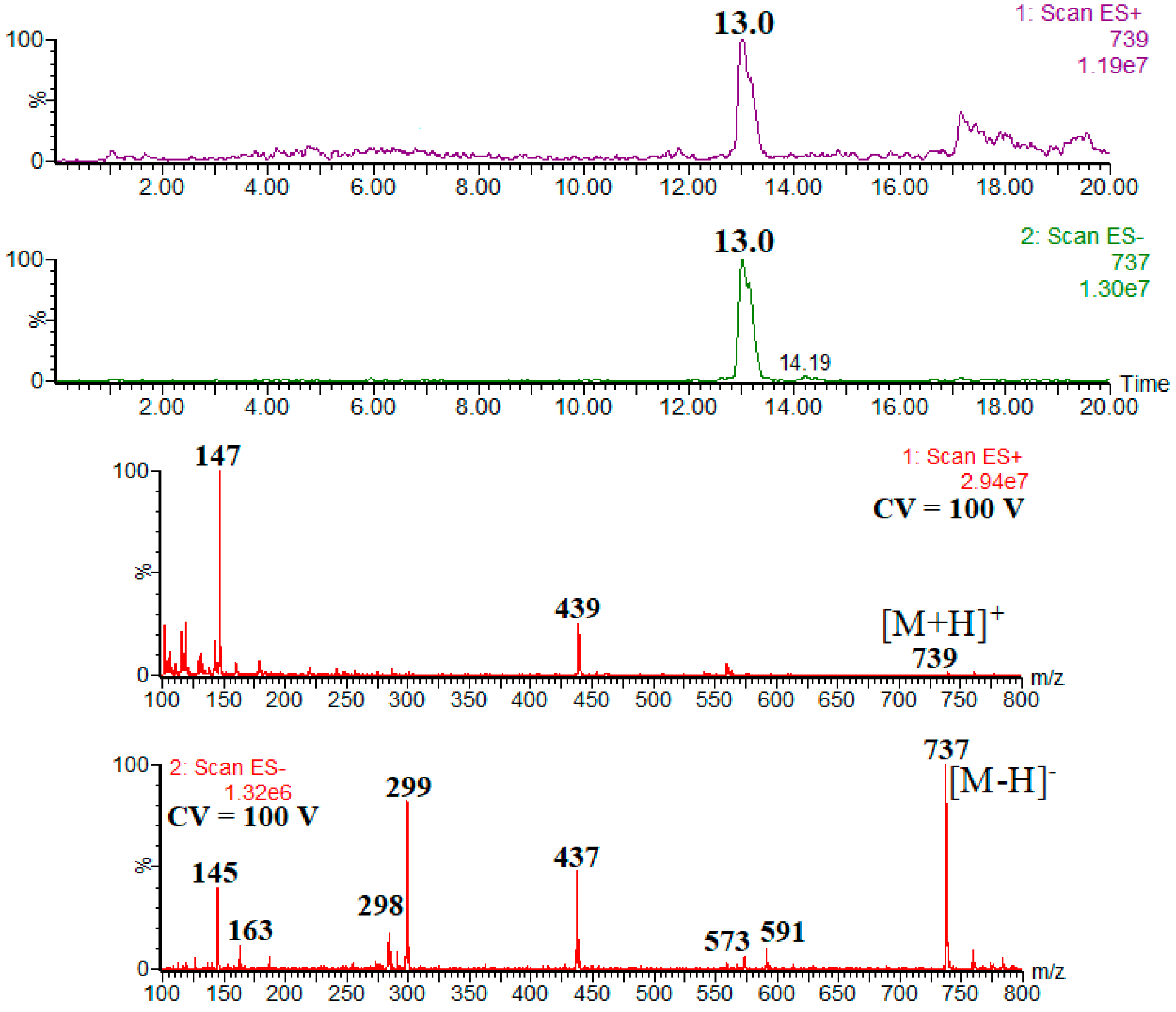

Figure S7. Single ion chromatograms of m/z 739 and 737, and ESI mass spectra of 4'-O-methyl-(2″,4″-di-E-*p*-coumaroyl)afzelin.

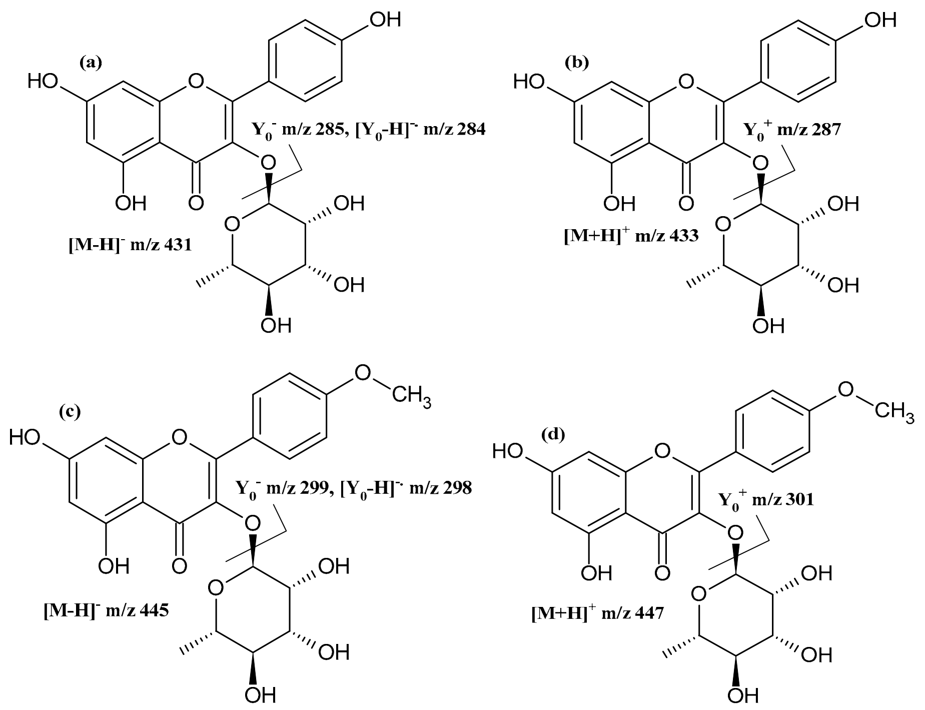

Scheme S1. The fragmentation pathways of $[M+H]^+$ and $[M-H]^-$ ions of afzelin (**a** and **b**) and 4'-O-methylafzelin (**c** and **d**).

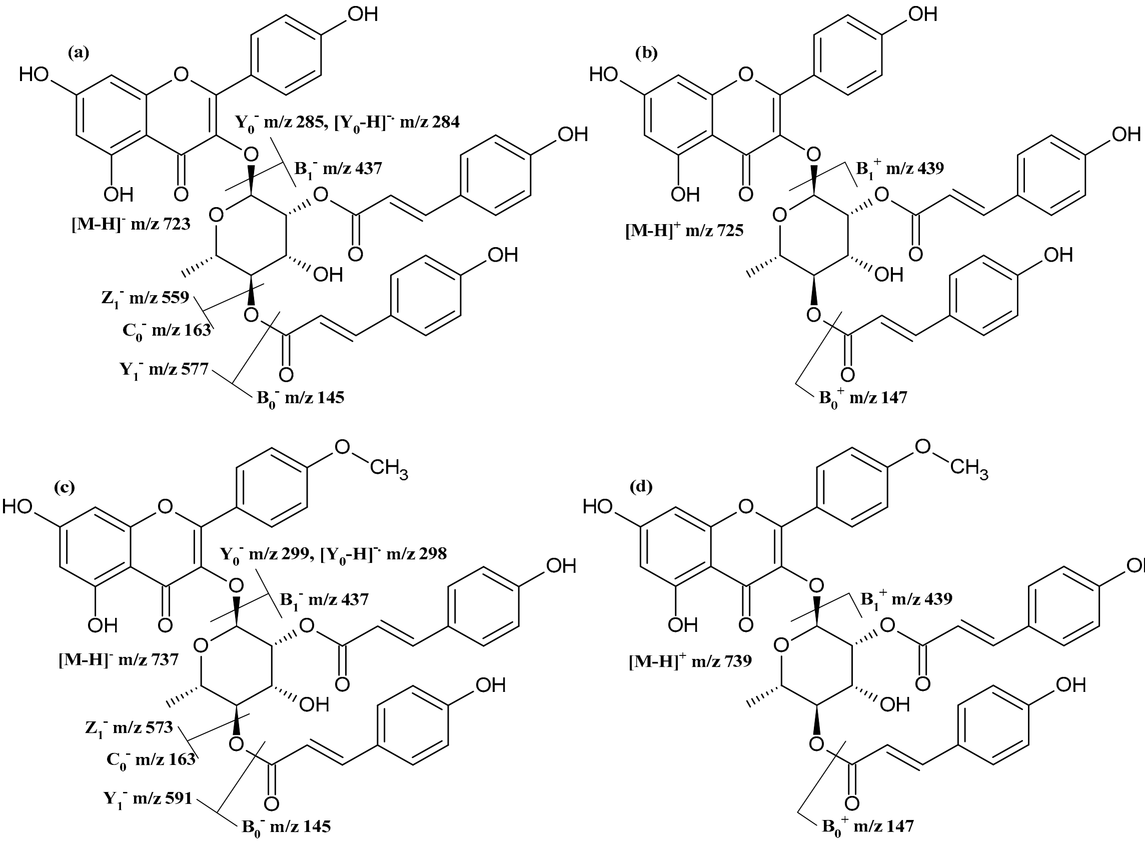

Scheme S2. The fragmentation pathways of $[M+H]^+$ and $[M-H]^-$ ions of (2″,4″-di-E-*p*-coumaroyl)afzelin (**a** and **b**) and 4’-O-methyl-(2″,4″-di-E-*p*-coumaroyl)afzelin (**c** and **d**).

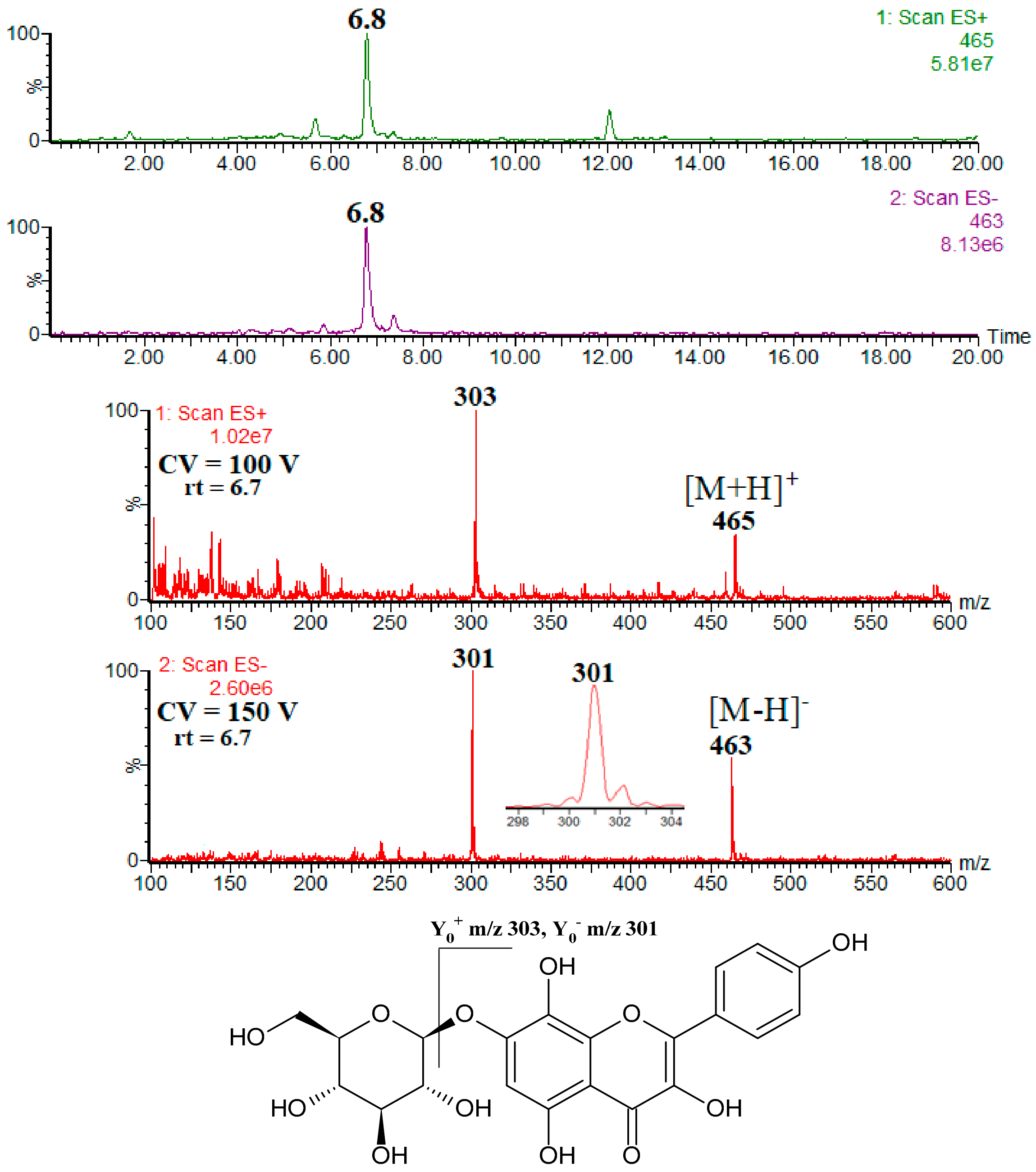

Figure S8. HPLC-MS data and observed fragmentation pathways of herbacetin 7-O-glucoside.

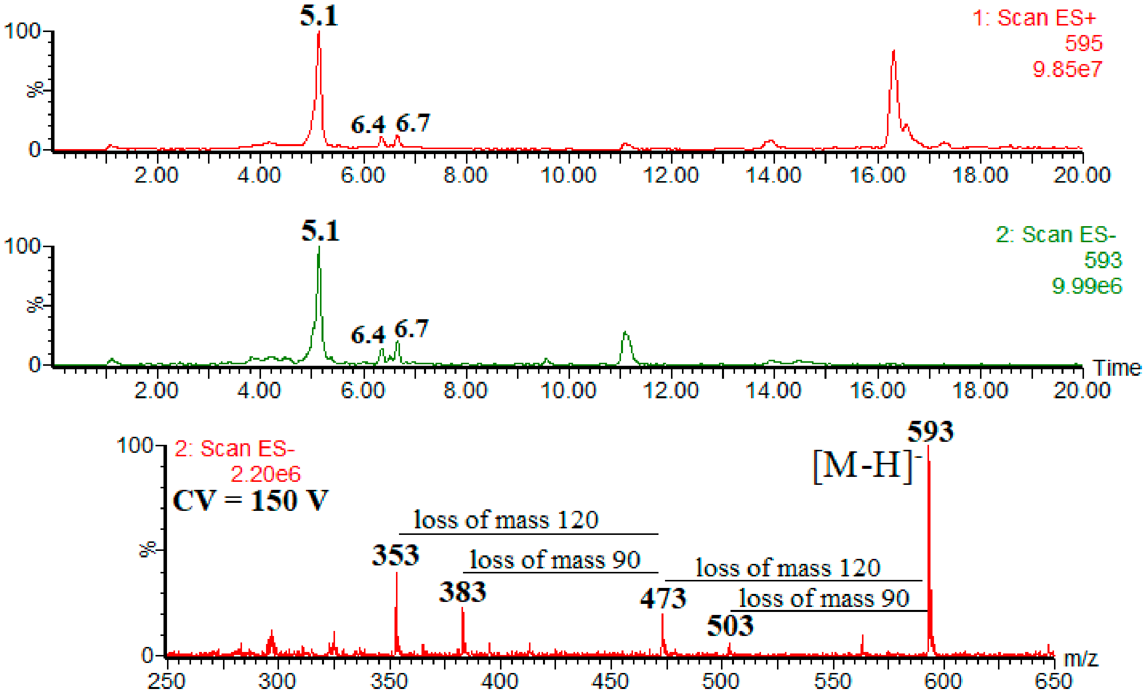

Figure S9. HPLC-MS data of the compound identified as vicenin-2.

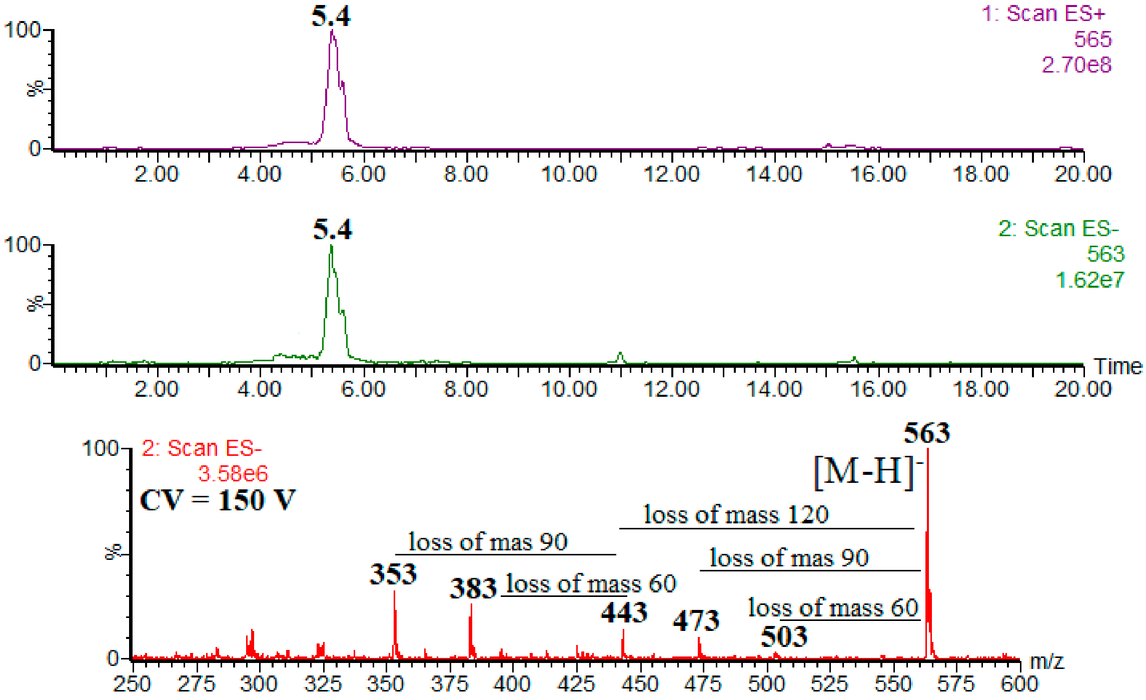

Figure S10. HPLC-MS data of the compound identified as schaftoside.

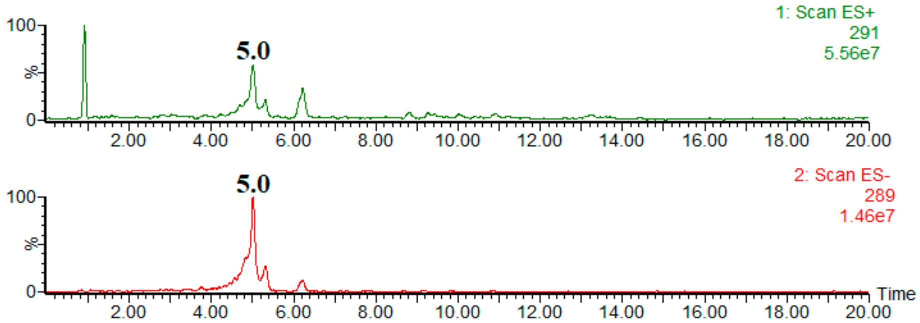

Figure S11. HPLC-MS identification of the catechin.
